# Water from Food in Young Chinese Adults: Patterns, Determinants, and Public Health Implications: A Cross-Sectional Study Across the Seven Geographic Regions

**DOI:** 10.3390/foods15010029

**Published:** 2025-12-22

**Authors:** Shuyi Zhou, Jianfen Zhang, Xiuhua Shen, Yu Wang, Meilin Zhang, Yong Jia, Wenli Zhu, Na Zhang, Guansheng Ma

**Affiliations:** 1 Department of Nutrition and Food Hygiene, School of Public Health, Peking University, Beijing 100191, China; 2 Department of Student Nutrition, National Institute for Nutrition and Health, Chinese Center for Disease Control and Prevention, 27 Nanwei Road, Xicheng District, Beijing 100050, China; 3 School of Medicine, Shanghai Jiao Tong University, Shanghai 200025, China; 4 School of Public Health, Lanzhou University, Lanzhou 730000, China; 5 School of Public Health, Tianjin Medical University, Tianjin 300070, China; 6 School of Nursing, Jilin University, Changchun 130021, China; 7 Laboratory of Toxicological Research and Risk Assessment for Food Safety, Peking University, Beijing 100191, China

**Keywords:** water from food, total water intake, hydration, dietary factors, young adults

## Abstract

Adequate hydration is essential for health, yet the contribution of water from food (WFF) to total water intake (TWI) and its determinants remain unclear in China. This study quantified WFF and explored factors influencing its variation among young Chinese adults. A multicentre cross-sectional survey was conducted in May–June 2023 among 947 healthy adults aged 18–25 years from seven regions of China. WFF was measured using three-day duplicate food portions, and drinking fluid intake was recorded with a validated 24 h diary. Sociodemographic, dietary, behavioral, and psychological data were collected using standardized instruments. Multivariable linear regression, stratified by sex and age, examined associations with WFF and its proportion of TWI. On average, WFF accounted for 38.0% of TWI, with regional variation from 33.5% in southern China to 44.3% in eastern China. Higher daily intake of energy, salt, and carbohydrate intakes were each positively associated with greater WFF, while carbohydrate intake seemed to betas the strongest predictor of a higher proportion of WFF. Younger age and elevated anxiety showed modest independent associations. These findings indicate that WFF contributes substantially to hydration in Chinese young adults, primarily driven by dietary composition. Recognizing WFF in hydration guidelines could improve population assessments and inform evidence-based nutrition strategies in China and other high-moisture diet regions.

## 1. Introduction

Water was formally recognized as an essential nutrient by the World Health Organization in 2005, emphasizing its fundamental role in maintaining life and physiological homeostasis [1]. Water is an essential nutrient critical for maintaining physiological functions, including thermoregulation, nutrient transport, waste elimination, and joint lubrication [2]. Adequate water intake is associated with improved health outcomes, including reduced risk of kidney stones, better cognitive performance, and potential prevention of obesity and cardiovascular disease [3].

Despite its fundamental importance, inadequate water intake remains highly prevalent worldwide. In the United States, only around 40% of adults achieve the Dietary Reference Intakes for total water [4]. Across Europe, 37% of men and 22% of women consume less than the levels recommended by the European Food Safety Authority [5,6]. Multicounty analyses further demonstrate pronounced geographic variation in TWI, shaped by climatic, dietary, and cultural factors. Hotter and more humid climates increase thermoregulatory water losses, resulting in higher TWI requirements. Dietary patterns, such as high consumption of soups, teas, or water-rich foods, further modulate fluid intake, while cultural practices, including habitual beverage choices and social drinking norms, contribute additional variability [5]. China faces a similar concerning situation. National and city-level surveys indicate that more than half of adults, and up to three-quarters of children and adolescents, fail to meet adequate intake levels [7]. Among young adults, approximately 55~80% do not achieve the recommended TWI [8]. These findings reveal a persistently high prevalence of inadequate hydration globally and within China, addressing the urgent need for context-specific, population-based strategies to promote adequate water intake.

Our findings align with cross-national evidence from the Western Pacific region, where populations consuming high-moisture diets, such as in China, Japan, and Korea, obtain a substantial portion of water from foods (WFF) [9,10]. In China, drinking fluid intake (DFI) accounts for around 60% of TWI, with WFF contributing roughly 38–40% [11]. Similarly, in Japan and Korea, WFF contributes approximately 35–50% of total water intake [9,10], compared with only 20–30% in most Western countries [12,13]. These data highlight that food-derived water is a meaningful component of total intake in East Asian diets and should not be overlooked in the development of hydration guidelines, public health campaigns, and nutrition policies aimed at promoting adequate water intake [9,10].

Research on the distribution and determinants of TWI remains limited, and evidence concerning WFF is even more scarce. Few studies have systematically examined how demographic, behavioral, and environmental factors influence WFF. However, these factors are likely to play a key role in shaping both the quantity and variability of WFF across populations [9,14,15,16]. Moreover, most available data rely on food frequency questionnaires or 3-day dietary recalls, which are likely to cause recall bias. Addressing these evidence gaps is essential for developing culturally appropriate, adequate water intake guidelines and public health policies, particularly in the Western Pacific region, where food-derived water constitutes a vital component of adequate water intake.

This study aims to quantify the contribution of WFF to TWI among young Chinese adults. It further seeks to identify key demographic, dietary, behavioral, and environmental determinants of WFF. By providing nationally representative evidence, the study intends to inform tailored hydration recommendations and support population-level strategies to improve water intake and overall hydration in East Asia.

## 2. Materials and Methods

### 2.1. Study Design

We conducted a multicenter, cross-sectional study from May to June 2023, targeting university students aged 18–25 years across China’s Seven Major Geographic Regions [17]. To ensure broad national representation, a multistage stratified sampling strategy was employed, and one university was selected in each region to capture variations in climate and sociocultural contexts: Tianjin Medical University (North), Jilin University (Northeast), Shanghai Jiao Tong University (East), Hainan Medical University (South), Yunnan Agricultural University (Southwest), Lanzhou University (Northwest), and Central South University (Central). Ethical approval was granted by the Peking University Ethical Review Committee (IRB00001052-22119), all procedures were conducted in accordance with the Declaration of Helsinki, and written informed consent was obtained from all participants.

The sample size was determined using the formula for cross-sectional studies:

(1) $$n=\frac{Z^{2}\times p\times \left(1-p\right)}{E^{2}},$$

where *n* is the sample size, *Z* is the Z statistic corresponding to a 95% confidence level (1.96), *p* is the expected prevalence of adults meeting the adequate water intake (0.551) [18], and *E* is the margin of error (0.09). The estimated base sample size per region was 118 participants. Allowing for a 10% non-response rate, the final target was 132 participants per region.

### 2.2. Participants and Recruitment

Participants were healthy undergraduate or graduate students aged 18–25 years. Exclusion criteria included recent use of drugs or dietary supplements, pregnancy or lactation, and chronic conditions affecting the digestive system, renal function, metabolism, or mental health. Recruitment combined online and on-site strategies: digital invitations were disseminated via social media platforms, including WeChat and QQ, while on-campus efforts involved informational posters, dedicated recruitment booths, and brief classroom presentations. Healthy status was assessed through self-reported medical history, current medications, and absence of chronic conditions. Although the total number of students approached and the exact response rate cannot be determined, these strategies were designed to maximize reach and encourage diverse participation. All promotional materials neutrally described the study objectives, procedures, and eligibility criteria to encourage broad and diverse participation.

### 2.3. Measurements

All data collection procedures strictly followed standardized protocols to ensure methodological consistency across study sites. Qualified investigators conducted all assessments using calibrated instruments and unified operational manuals. On Day 1, participants underwent anthropometric assessments, including height and body weight, measured by trained personnel with standardized techniques. Subsequently, participants were instructed and supervised to complete a 7-day, 24 h fluid intake diary documenting all beverages consumed. Investigators reviewed and verified logs daily to ensure completeness and accuracy. During three consecutive dietary monitoring days (Days 5–7), including two weekdays and one weekend day. All foods consumed were weighed and recorded in real time by trained study investigators using electronic scales in the designated university cafeterias. Dietary intake was assessed for three consecutive days to represent habitual consumption while minimizing participant burden. Fluid intake was monitored for seven consecutive days to capture typical daily hydration patterns accurately. In parallel, 24 h urine samples (including the first morning void) were collected each day for sodium analyses. Throughout the observation period, participants were instructed to maintain habitual routines and reside under stable environmental conditions to minimize behavioral and external variability.

#### 2.3.1. Anthropometric Measurements and Questionnaire Assessment

Trained researchers performed duplicate post-baseline anthropometric measurements (weight and height) in the morning after an overnight fast and post-void, using standardized equipment. Height and weight were measured with digital devices (HDM-300, Huaju, Zhejiang, China; AccuMeasure, Greenwood Village, CO, USA) and recorded to the nearest 0.1 cm and 0.1 kg, respectively. Socioeconomic position was estimated using the Chinese City Tier System [19], which classifies urban centers into first-tier, emerging first-tier, second-tier, and third-tier categories based on economic vitality, transit systems, built environment, and cultural prominence [20]. This approach provides a practical, region-level indicator of socioeconomic context.

Participants completed structured questionnaires, including demographic and socioeconomic information, the International Physical Activity Questionnaire (IPAQ) [21,22], the Pittsburgh Sleep Quality Index (PSQI) [23], and standardized psychological instruments, including the Self-Rating Anxiety Scale (SAS) [24] and Self-Rating Depression Scale (SDS) [25]. Study personnel supervised questionnaire administration to ensure accurate and complete responses.

#### 2.3.2. Water Intake Assessment

TWI was calculated as the sum of DFI and WFF. To assess DFI, participants completed a validated 7-day, round-the-clock self-reported beverage log following a brief practice period [26]. The log captured detailed information on beverage types, volumes, and consumption settings. All liquids, including bottled water, sweetened beverages, and dairy, were measured using team-provided cups calibrated to 5 mL accuracy. Trained staff reviewed the logs daily to ensure completeness and accuracy.

WFF was quantified using the duplicate-portion sampling method, coupled with oven-drying of the collected samples to constant weight over three consecutive days (two weekdays and one weekend day), consistent with established nutritional survey recommendations for capturing short-term variability while minimizing participant burden. Meals were primarily consumed at designated campus dining facilities. For each participant, all foods consumed were weighed before and after consumption to capture individual intake, with a precision of 0.1 g. For each unique dish, a single representative duplicate portion was collected for laboratory analysis to determine moisture content. Immediately after collection, duplicate samples were placed in 4 °C portable coolers, transferred to −20 °C freezers, and subsequently transported via −20 °C cold-chain to a central laboratory. Moisture from food was determined by oven drying at 105 °C until constant weight, in accordance with China’s GB 5009.3-2016 protocol [27]. Water from supplementary items, such as fruits or snacks, was calculated by trained personnel using values from the 2009 Chinese Food Composition Tables [28].

To maintain ecological validity, foods consumed outside the canteen—including restaurant meals, convenience-store items, and takeout—were also incorporated. Participants weighed these foods before and after consumption using the provided digital scales and obtained an identical portion of each unique dish for duplicate sampling. They were additionally asked to provide an identical portion of each unique dish for duplicate sampling. These samples were handled identically to on-campus foods, ensuring consistent laboratory-based assessment of water content across all food sources.

#### 2.3.3. Dietary Intake Assessment

Building on the three-day sampling protocol for WFF, all foods consumed by participants were accurately recorded. Daily average energy was estimated by converting measured food weights (grams) using nutrient composition values per 100 g from validated Chinese food composition databases. Energy content was calculated according to GB 28050-2011 [29]; protein concentrations followed GB 5009.5-2016 (Determination of Protein in Foods (Method 1)) [30]. Daily salt intake (g) was estimated from complete 24 h urine collections using the validated formula adapted from a previous study [31]:

(2) $$\mathrm{Salt}\;\left(g\right)=\frac{100\times 2.54\times 23\times 24\;h\;\mathrm{urinary}\;\mathrm{Na}\;(mmol/L)\times 24\;h\;\mathrm{volume}\;(L)}{1}$$

Participants recorded all voids using graduated containers, and trained staff supervised collections when feasible and reviewed logs daily for completeness. Urinary sodium concentrations were measured using an automated biochemical analyzer with the ion-selective electrode potentiometric method (AU 5800; Beckman, Brea, CA, USA).

#### 2.3.4. Regional Environmental Exposure

Environmental parameters, including temperature and relative humidity, were systematically recorded throughout the study. Measurements were conducted three times daily (10:00, 14:00, and 20:00) for both indoor and outdoor settings using a calibrated temperature and humidity meter (WSB-1-H2; Exasace, Suzhou, China), providing accuracy of ±1 °C for temperature and ±0.1% for relative humidity. In addition, ambient conditions at participants’ residences were logged each day at 10:00 AM to capture variations in home environments over the study period.

### 2.4. Quality Control

A comprehensive quality assurance framework was implemented to ensure procedural consistency of collected data. All investigators completed standardized training and certification, and field procedures followed a detailed operations manual. Anthropometric measurements were performed using calibrated equipment under direct supervision, with duplicate measurements required to fall within <0.2 cm for height and <0.2 kg for weight.

To improve fluid intake assessment, participants maintained a validated seven-day beverage diary with 5 mL resolution. Investigators conducted daily reviews of diaries and immediately verified unclear or missing entries with participants, resulting in <2% entries removed due to unresolved missing data. Snack intake was documented through participant photographs and was verified by investigators, with weighing performed when applicable. Nutrient content of consumed foods was determined using the duplicate-portion method in accordance with national guidelines. For biological samples, <2% of 24 h urine collections were rejected due to incompleteness, verified through participant logs and investigator supervision. Collected specimens were stored at −20 °C, transported under cold-chain conditions (−20 °C), and analyzed centrally to limit inter-laboratory variability.

Data management incorporated a double-entry system with independent verification and automated logic checks to identify missing or inconsistent entries, which were promptly addressed. Protocol adherence exceeded 90%, and any deviations were documented and corrected in real time. Personal identifiers were replaced with anonymized codes, and electronic records were secured on encrypted servers with role-based access control.

### 2.5. Statistical Analysis

Descriptive analyses were conducted to characterize the study cohort and summarize beverage consumption patterns across geographic regions and participant subgroups. Normality of continuous variables was evaluated using the Kolmogorov–Smirnov test. Given the predominance of non-normally distributed variables, medians and interquartile ranges (IQRs) are reported. Comparisons between two groups were performed with the Mann–Whitney U test, whereas the Kruskal–Wallis test was applied for comparisons involving more than two groups; Dunn’s post hoc procedure was used for pairwise contrasts when overall differences reached statistical significance. Categorical variables are presented as counts and percentages and compared using χ^2^ tests or Fisher’s exact tests, as appropriate. Missing data were not imputed, and analyses were conducted using available cases only.

To examine factors associated with WFF intake, multivariable linear regression models were constructed with daily WFF volume as the outcome. Covariates were selected a priori based on theoretical rationale and prior evidence and included demographic and socioeconomic indicators (age, sex, ethnicity, city-tier classification, and region), dietary intake (total energy high fat intake, high protein intake, high carbohydrate intake, salt), lifestyle factors (physical activity from IPAQ, sleep quality via PSQI), and psychological measures (SAS and SDS scores). Regression outcomes are expressed as β coefficients with 95% confidence intervals (CIs) and corresponding *p*-values. Macronutrient thresholds were defined according to national dietary reference cutoffs: high protein (>65 g/day for men, >55 g/day for women), high carbohydrate (>150 g/day), and high fat (>30% of total energy) [2].

Subgroup analyses stratified by sex and age (18–19, 20–21, and 22–25 years) were performed to explore potential heterogeneity in beverage consumption and its determinants. Fully adjusted models were re-estimated within each subgroup. Interaction terms between beverage categories and sex or age were included to formally assess effect modification, and the significance of these interactions was evaluated.

All analyses were carried out using R software (version 4.3.2; R Foundation for Statistical Computing, Vienna, Austria). Statistical significance was defined as two-sided *p* < 0.05, with *p*-values between 0.05 and 0.10 considered borderline significant.

## 3. Results

### 3.1. Characteristics of Participants

**Figure 1:**
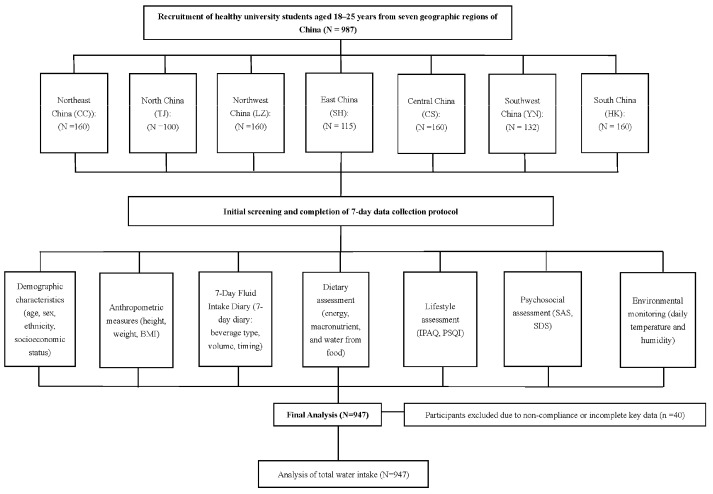
Flow diagram of study participants. A total of 987 healthy university students aged 18–25 years were recruited from seven geographic regions of China. After the 7-day data collection protocol, 40 participants were excluded due to non-compliance or incomplete data, resulting in a final sample of 947 participants. BMI = Body Mass Index. IPAQ = International Physical Activity Questionnaire. PSQI = Pittsburgh Sleep Quality Index. SAS = Self-Rating Anxiety Scale. SDS = Self-Rating Depression Scale.

A total of 947 healthy young adults were included in this cross-sectional analysis. The recruitment and inclusion process is presented in Figure 1, and participant characteristics are summarized in Table 1. The median age was 20.0 years (IQR 19.0–21.0). Females accounted for 53.4% (n = 504) and males for 46.6% (n = 440). The majority were of Han ethnicity (85.2%, n = 807), while 14.8% (n = 140) were from ethnic minority groups. With respect to body composition, 16.7% (n = 150) were underweight, 64.4% (n = 578) had normal weight, 14.5% (n = 130) were overweight, and 4.5% (n = 40) were classified as obese.

Participants were recruited from seven geographically diverse regions of China: Northeast (Changchun, 16.9%, n = 160), North (Tianjin, 10.0%, n = 95), Northwest (Lanzhou, 16.7%, n = 158), East (Shanghai, 10.7%, n = 101), Central (Changsha, 16.8%, n = 159), Southwest (Yunnan, 12.4%, n = 117), and South (Haikou, 16.6%, n = 157). Socioeconomic classification based on the Chinese City Tier System indicated that 14.2% of participants were from Tier 1 cities, 28.6% from emerging Tier 1 (Tier 1.5), and 57.1% from Tier 2 cities.

Regarding lifestyle patterns, 15.9% (n = 150) had low physical activity, 38.9% (n = 367) moderate, and 45.2% (n = 427) high activity levels. The median PSQI score was 6.0 (IQR 4.0–8.0), indicating generally good sleep quality. Median daily energy intake was 1750 kcal/day (IQR 1442–2088), with median fat intake of 72.2 g/day (IQR 59.8–87.2), protein intake of 62.5 g/day (IQR 49.9–74.5), and carbohydrate intake of 218.4 g/day (IQR 172.8–267.1). Median salt intake was 6.48 g/day (IQR 4.62–8.66). Psychological assessments showed a median SAS score of 35.0 (IQR 32.0–38.0) and a median SDS score of 43.0 (IQR 39.3–47.0).

For water intake, the median TWI was 2117.9 mL/day (IQR 1753.3–2575.3), composed of 1259.9 mL/day (IQR 938.9–1593.0) from DFI and 835.9 mL/day (IQR 667.1–1005.2) from WFF.

To contextualize regional environmental variability, we summarized indoor and outdoor temperature and humidity conditions for all seven study regions (Supplementary Table S1).

### 3.2. WFF Distribution and Regional Disparities

**Figure 2:**
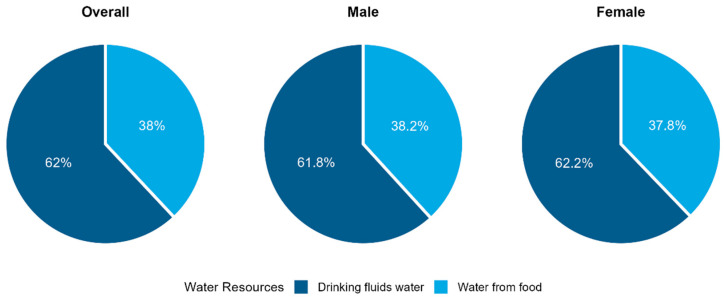
Proportion of water sources in total daily water intake by sex. Pie charts show contributions from drinking fluids water (dark blue) and water from food (light blue) in the overall population and separately for males and females.

**Figure 3:**
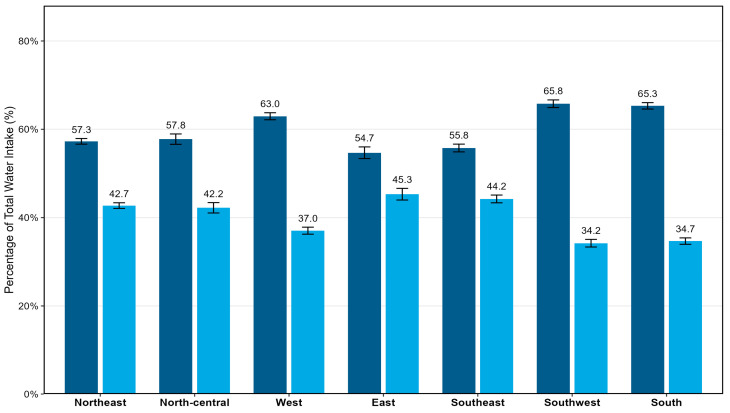
Contribution of DFI and WFF to TWI by region. Bars represent mean percentage contribution of DFI (dark blue) and WFF (light blue) to TWI. Data are expressed as Mean ± Standard Error (SE). Regions are displayed from Northeast to South China: Northeast China (Changchun, CC), North China (Tianjin, TJ), Northwest China (Lanzhou, LZ), East China (Shanghai, SH), Central China (Changsha, CS), Southwest China (Yunnan, YN), and South China (Haikou, HK).

Overall, WFF accounted for 38.0% of TWI, with the remaining 62.8% derived from DFI. Sex-specific differences were minimal: women obtained 37.8% of WFF and men 38.2%, indicating a comparable relative contribution of food moisture across sexes (Figure 2). Substantial regional variability in WFF proportion was observed (Figure 3). Across seven regions, the contribution of WFF to TWI ranged from 34.2% in Southwest China (Yunnan, YN), 34.7% in South China (Haikou, HK), and 37% in West (Lan Zhou, LZ) to 45.3% in East China (Shanghai, SH; *p* < 0.001). Sex differences within regions were similar.

### 3.3. Overall Determinants of WFF

Building on the observed regional variability, we further explored individual-level determinants of WFF across the full cohort (Table 2). Dietary factors emerged as the predominant contributors, whereas age and anxiety modestly influenced the %WFF. Higher daily energy intake (β = 0.183, 95% CI 0.140–0.227, *p* < 0.001), salt intake (β = 14.657, 95% CI 9.954–19.359, *p* < 0.001), and carbohydrate intake (β = 228.169, 95% CI 181.135–275.203, *p* < 0.001) were all positively associated with greater WFF volume. In contrast, only carbohydrate intake showed a significant association with %WFF (β = 0.081, 95% CI 0.060–0.102, *p* < 0.001). Among non-dietary factors, older age predicted a lower %WFF (β = −0.008, 95% CI −0.012 to −0.003, *p* = 0.003), while higher anxiety levels were linked to a slightly higher %WFF (β = 0.001, 95% CI 0.000–0.002, *p* = 0.047). No significant associations were identified for sex, ethnicity, physical activity, depressive or sleep symptoms, or regional meteorological variables.

### 3.4. Sex-Stratified Analysis

In sex-stratified analyses (Supplementary Table S2), dietary determinants of WFF remained consistent in both sexes, though the magnitudes differed. Energy and carbohydrate intake exerted stronger effects in men, whereas salt intake was more influential in women. Specifically, daily energy intake was associated with higher WFF in men (β = 0.230, 95% CI 0.170–0.290, *p* < 0.001) and women (β = 0.099, 95% CI 0.033–0.165, *p* = 0.003); salt intake remained significant in both (men: β = 11.159, 95% CI 4.860–17.458, *p* = 0.001; women: β = 19.349, 95% CI 12.285–26.412, *p* < 0.001). Carbohydrate intake consistently predicted higher WFF and %WFF in both men and women, with effect estimates larger in men (β = 346.953) than in women (β = 193.069, both *p* < 0.001). Anxiety (SAS score) was associated with a higher %WFF in men (β = 0.002, 95% CI 0.000–0.003, *p* = 0.038), whereas age predicted a lower %WFF in women (β = −0.008, 95% CI −0.014 to −0.001, *p* = 0.028).

### 3.5. Age-Stratified Analysis

When stratified by age group, dietary effects persisted, particularly among younger adults (Supplementary Table S3). Daily energy intake was positively associated with WFF across all age groups, most strongly among those aged 18–19 years (β = 0.277, 95% CI 0.211–0.343, *p* < 0.001), with weaker associations in 20–21 years (β = 0.129, *p* < 0.001) and 22–25 years (β = 0.152, *p* = 0.020). Salt intake showed a similar positive pattern in younger groups, with β = 13.627 (*p* < 0.001) for 18–19 years and β = 15.042 (*p* < 0.001) for 20–21 years. High carbohydrate intake was consistently linked to greater WFF and %WFF, particularly in the youngest adults (β = 0.101, *p* < 0.001 for 18–19 years; β = 0.066, *p* < 0.001 for 20–21 years). Among psychosocial variables, higher anxiety was associated with higher %WFF (β = 0.002, *p* = 0.025), whereas poorer sleep quality (PSQI) correlated with lower %WFF (β = −0.004, *p* = 0.037), both effects limited to the youngest subgroup.

## 4. Discussion

### 4.1. Principal Findings

In this nationally and regionally representative study of young Chinese adults, WFF contributed substantially to TWI (about 40%), with substantial regional variations. Dietary factors, particularly total energy, sodium, and carbohydrate intake, emerged as the predominant determinants of WFF, whereas age and anxiety levels primarily influenced %WFF.

These findings provide nationally representative evidence on food-derived water intake, supporting the development of culturally and contextually appropriate recommendations for adequate water intake in China. They also have broader relevance for other countries in the Western Pacific region, where traditional diets are typically high in water-rich foods such as soups, fruits, and vegetables.

### 4.2. Comparison with Previous Studies and Possible Explanations

Our findings are consistent with observations in other East Asian populations, where WFF accounts for roughly 35–60% of TWI [8,9,11,32], substantially higher than the proportions reported in Western countries, typically about 20~30% [4]. These differences can be attributed to dietary composition: East Asian diets often emphasize water-rich staples like rice and noodles, soups, vegetables, and fruits, which inherently boost WFF through direct water content and cooking methods that retain moisture [9]. In contrast, Western diets rely more on lower-moisture foods like bread and processed items, leading to greater dependence on beverages for hydration [4]. For example, in the United States, only about 23% of TWI came from food moisture, indicating a predominance of beverage-derived hydration in this Western dietary context [4]. In addition, national surveys in multiple countries, such as France and the UK, show that food moisture contributes roughly 27–36% of TWI [6], consistent with other European samples where fluids dominate total water intake [5].

Potential explanations for East–West differences in WFF contributions. Water content of foods varies substantially by food type and overall dietary pattern. Plant-based foods such as fruits and vegetables, common in many Asian diets, typically contain very high water proportions (~80–95%), and liquid or broth-based foods like soups also have high moisture, whereas many Western staples and processed foods have much lower water content (<15% in dry products) [33]. Cooking methods influence the water retained in foods: practices common in East Asian cuisines, such as steaming, boiling, or stewing, tend to preserve or add water to dishes, while Western methods like baking or frying do not contribute additional water and may reduce inherent moisture, affecting the amount of food moisture available for hydration [33].

Within China, regional variation likely reflects differences in dietary patterns, cooking practices, cultural habits, and environmental factors such as climate and urbanization [10,11,34]. Populations in Eastern China, where soups, vegetables, and fruits are consumed more frequently, exhibited higher WFF, whereas those in Western China, characterized by greater reliance on staple grains and lower-moisture foods, showed lower WFF. In Southern China, particularly in Haikou, lower WFF was partly offset by high consumption of local beverages such as coconut water, contributing substantially to total water intake. Seasonal variation also may affect WFF and TWI in China; prospective cohort data show that TWI and its composition (including WFF and DFI) differ significantly across seasons, with higher TWI in warmer seasons than cooler ones, indicating that environmental temperature and seasonality modulate overall water intake patterns [11].

Higher total energy intake was strongly associated with WFF, suggesting that greater food volume consumption inherently provides more water, consistent with energy-driven eating behaviors observed in prior surveys [9,12]. Salt intake also showed a positive association with WFF, consistent with population studies in UK and Australian children demonstrating that higher sodium exposure is reliably accompanied by increased fluid intake. Mechanistically, elevated sodium concentration may raise plasma osmolality, activating central osmoreceptors within the hypothalamus and triggering thirst responses aimed at restoring osmotic balance, thereby increasing fluid consumption [35]. Among macronutrients, carbohydrate intake independently predicted WFF, likely due to the high-water content of carbohydrate-rich foods.

Beyond diet, psychological and lifestyle factors influenced relative WFF contribution. Anxiety symptoms were associated with variation in %WFF, whereas poor sleep quality corresponded to lower relative WFF, suggesting that behavioral rhythms and meal patterns may indirectly affect adequate water intake [36,37]. Previous studies found that anxiety and depression in college students were linked to altered food choices, such as higher sugar and lower fruit/vegetable intake, which could indirectly affect water consumption from foods [37]. Similarly, better sleep quality has been associated with higher plain water intake and improved hydration status, while experimental data indicate that fluid intake correlates with REM sleep duration and sleep efficiency [38].

### 4.3. Strengths, Limitations, and Future Directions

This is the first nationally and regionally representative multicenter study examining determinants of WFF among healthy young Chinese adults. By including seven geographically diverse sites, we captured variation in dietary habits, climate, and urbanization, allowing a comprehensive assessment of population-level determinants.

A key strength of this study lies in the precise quantification of WFF using a three-day duplicate diet combined with drying methods, which substantially reduces measurement error compared with traditional dietary assessment methods such as food frequency questionnaires, 24 h recalls, or short-term food records. DFI was concurrently assessed through seven-day validated questionnaires with calibrated measurement cups, providing more accurate estimates of beverage-derived water. The integration of these rigorously collected data ensures highly reliable estimates of TWI and the proportion of %WFF, allowing robust evaluation of both absolute and relative contributions of food-derived water across regions and dietary patterns.

Despite these strengths, several limitations must be acknowledged. The cross-sectional design precludes causal inference. Findings are restricted to young adults aged 18–25 years, and data were collected only in May–June from a single university per region, which may limit generalizability to other age groups, seasons, and institutional settings. Despite the multi-region design offering some insight into climatic variability, certain behavioral and dietary factors were not exhaustively captured, and residual confounding may still exist. Behavioral and demographic data were self-reported, introducing potential measurement bias. Moreover, the prevalence employed for sample-size determination was informed by prior studies of college students and might differ from national averages. While analyses considered sex differences, the study did not capture social or cultural dimensions of gender, which may influence dietary and hydration behaviors.

Future studies should employ longitudinal or interventional designs to establish causal relationships and explore additional determinants, including social, cultural, and seasonal influences. Extending research to broader age ranges and other high-moisture diet populations in the Asia-Pacific region would further refine understanding of adequate water intake patterns and inform evidence-based, culturally appropriate guidance for food-derived intake.

### 4.4. Public Health Implications

These findings highlight the importance of considering food-derived water when evaluating adequate water intake, particularly in countries with high-moisture diets. Current hydration guidelines focus primarily on beverage consumption, potentially underestimating the importance of WFF. Policymakers should consider region-specific, age- and sex-sensitive recommendations that reflect local culinary practices and demographic differences in water intake. For example, populations in eastern regions already consume substantial water through food, whereas western populations may benefit from targeted interventions to increase WFF through culturally appropriate dishes. Implementation can be supported through institutional and community-based programs, including school meal planning, workplace wellness initiatives, and nutrition curricula that address both fluid and food sources of water. Such approaches operationalize the evidence and offer practical, low-cost pathways to strengthen population status at the population level. From a guideline perspective, explicitly recognizing the contribution of food moisture in national hydration recommendations could support more accurate assessment of hydration adequacy and reduce reliance on beverage intake alone, particularly in contexts where food provides a substantial share of TWI.

## 5. Conclusions

In this nationally representative sample of young Chinese adults, WFF accounted for approximately 38–40% of total water intake, underscoring its substantial contribution to hydration. WFF intake was primarily determined by dietary factors, particularly total energy, sodium, and carbohydrate intake, alongside modest influences from age, psychological characteristics, and regional dietary patterns. These findings highlight the need for hydration guidance that integrates both beverages and food-derived water and reflects demographic and regional variability in dietary practices. Future research should incorporate seasonal variation, longitudinal assessments, and intervention-based approaches to more fully characterize determinants of food-derived water intake and to inform evidence-based hydration strategies for young adults in China and East Asia.

## Figures and Tables

**Table 1:** Characteristics of participants.

Variable	Overall (N = 947)
Demographic characteristics	
Age (years)	20.000 (19.000, 21.000)
Ethnicity, n (%)	
Han	807 (85.2%)
Minority	140 (14.8%)
Sex, n (%)	
Male	440 (46.6%)
Female	504 (53.4%)
BMI category, n (%)	
Underweight	150 (16.7%)
Normal weight	578 (64.4%)
Overweight	130 (14.5%)
Obesity	40 (4.5%)
Region, n (%)	
Northeast China (Changchun, CC)	160 (16.9%)
North China (Tianjin, TJ)	95 (10.0%)
Northwest China (Lanzhou, LZ)	158 (16.7%)
East China (Shanghai, SH)	101 (10.7%)
Central China (Changsha, CS)	159 (16.8%)
Southwest China (Yunnan, YN)	117 (12.4%)
South China (Haikou, HK)	157 (16.6%)
Socioeconomic status	
Tier (1)	1 (14.2%)
Tier (1.5)	2 (28.6%)
Tier (2)	4 (57.1%)
Lifestyle factors	
Physical activity level, n (%)	
Low	150 (15.9%)
Medium	367 (38.9%)
High	427 (45.2%)
PSQI score	6.000 (4.000, 8.000)
Diet	
Daily energy intake (kcal/day)	1750.025 (1442.138, 2087.717)
Daily fat intake (g/day)	72.190 (59.799, 87.168)
Daily protein intake (g/day)	62.466 (49.920, 74.504)
Daily carbohydrate intake (g/day)	218.390 (172.770, 267.055)
Daily salt intake (g/day)	6.480 (4.623, 8.655)
Psychological factors	
SAS score	35.000 (32.000, 38.000)
SDS score	43.000 (39.250, 47.000)
Water intake variables	
TWI (mL/day)	2117.856 (1753.251, 2575.298)
DFI (mL/day)	1259.910 (938.888, 1593.000)
WFF (mL/day)	835.945 (667.073, 1005.232)

Data are presented as median (interquartile range) for continuous variables and n (%) for categorical variables, unless otherwise specified. Participants were healthy young adults aged 18–24 years, recruited from seven regions across mainland China: Northeast (Changchun), North (Tianjin), Northwest (Lanzhou), East (Shanghai), Central (Changsha), Southwest (Yunnan), and South (Haikou). Socioeconomic status was classified according to the Chinese City Tier System: Tier 1 (major metropolitan centers), Tier 1.5 (emerging first-tier cities), and Tier 2 (developing urban centers). Body mass index (BMI) was categorized based on Chinese standards: underweight (<18.5 kg/m^2^), normal (18.5–23.9 kg/m^2^), overweight (24.0–27.9 kg/m^2^), and obese (≥28.0 kg/m^2^). PSQI, Pittsburgh Sleep Quality Index; SAS, Self-Rating Anxiety Scale; SDS, Self-Rating Depression Scale; TWI, total water intake; DFI, drinking fluid intake; WFF, water from food.

**Table 2:** Associations of demographic, psychosocial, lifestyle, and dietary factors with WFF intake and its contribution to total daily water intake in Chinese adults.

Variables	WFF		%WFF	
	β (95% CI)	*p*	β (95% CI)	*p*
Sex (Female vs. Male)	14.734 [−17.942, 47.410]	0.376	0.017 [0.002, 0.032]	**0.022**
Age (years)	5.444 [−5.313, 16.202]	0.321	−0.008 [−0.012, −0.003]	**0.003**
Ethnicity (non-Han)	10.836 [−30.140, 51.812]	0.604	0.013 [−0.006, 0.031]	0.172
Socioeconomic Tier (1.5)	−12.892 [−266.645, 240.86]	0.859	−0.032 [−0.281, 0.217]	0.642
Socioeconomic Tier (2)	−168.277 [−448.297, 111.743]	0.128	−0.092 [−0.364, 0.179]	0.283
PA Level (Moderate)	−8.666 [−52.136, 34.804]	0.696	0.000 [−0.020, 0.020]	0.997
PA Level (High)	15.598 [−26.507, 57.702]	0.467	−0.005 [−0.024, 0.014]	0.581
SAS Score	0.836 [−1.911, 3.584]	0.551	0.001 [0.000, 0.002]	**0.047**
SDS Score	−0.708 [−3.324, 1.907]	0.595	−0.001 [−0.002, 0.001]	0.266
PSQI Score	−2.223 [−8.244, 3.798]	0.469	−0.001 [−0.004, 0.002]	0.529
Daily Energy Intake (kcal/day)	0.183 [0.140, 0.227]	**<0.001**	0.000 [0.000, 0.000]	**<0.001**
Daily Salt Intake (g/day)	14.657 [9.954, 19.359]	**<0.001**	0.001 [−0.001, 0.003]	0.332
High Fat Intake	7.409 [−43.283, 58.101]	0.774	−0.021 [−0.044, 0.002]	0.072
High Protein Intake	3.809 [−36.716, 44.334]	0.854	−0.014 [−0.033, 0.004]	0.128
High Carbohydrate Intake	228.169 [181.135, 275.203]	**<0.001**	0.081 [0.060, 0.102]	**<0.001**
Region Temperature (°C)	−0.330 [−51.14, 50.48]	0.979	0.000 [−0.046, 0.046]	0.973
Region Relative Humidity (%)	−2.361 [−11.319, 6.596]	0.361	−0.001 [−0.009, 0.007]	0.655

β coefficients (95% confidence intervals) and *p*-values were estimated from multiple linear regression models examining the associations of sociodemographic, psychological, and dietary factors with absolute water from food (WFF) intake and its percentage of total water intake (%WFF). Models were adjusted for sex, age, ethnicity, socioeconomic tier, physical activity level, anxiety (SAS score), depression (SDS score), sleep quality (PSQI score), daily energy and salt intake, macronutrient composition, and regional temperature. Statistical significance was defined as *p* < 0.05 and bold.

## Data Availability

The data presented in this study are available on request from the corresponding authors. The data are not publicly available due to privacy and ethical restrictions.

## Supplementary Materials

The following supporting information can be downloaded at https://www.mdpi.com/article/10.3390/foods15010029/s1. Table S1: Regional indoor and outdoor temperature and humidity characteristics across seven Chinese cities; Table S2: Percentage contribution of water from food and drinking fluids to total water intake across seven geographical regions in China. Table S3: Associations of demographic, psychosocial, lifestyle, and dietary factors with water from food (WFF) intake and its percentage contribution to total water intake among Chinese adults. Table S4: Associations of demographic, psychosocial, lifestyle, and dietary factors with water from food (WFF) intake and its percentage of total water intake (%WFF) stratified by age group among Chinese adults.

## Abbreviations

The following abbreviations are used in this manuscript:

ChiCTR	Chinese Clinical Trial Registry
CI	Confidence Interval
DFI	Daily Fluid Intake
GB/T	Guobiao Standard (National Standard of China)
IPAQ	International Physical Activity Questionnaire
IQR	Interquartile Range
IRB	Institutional Review Board
PSQI	Pittsburgh Sleep Quality Index
SAS	Self-Rating Anxiety Scale
SDS	Self-Rating Depression Scale
TWI	Total Water Intake
WFF	Water From Food
WHO	World Health Organization
