# Supplementary material for: Water from Food in Young Chinese Adults: Patterns, Determinants, and Public Health Implications: A Cross-Sectional Study Across the Seven Geographic Regions
Source: Foods. 2025 Dec 22;15(1):29. doi: 10.3390/foods15010029 (PMC12785264; doi:10.3390/foods15010029)
Supplement: Supplementary file 1 [file foods-15-00029-s001.zip › foods-3984220-supplementary.pdf]

Supplementary Materials

Table S1. Regional indoor and outdoor temperature and humidity characteristics across seven Chinese cities

Region	Indoor Temperature (∘C)	Indoor Humidity (%)	Outdoor Temperature (∘C)	Outdoor Humidity (%)
Northeast China (Changchun, CC)	25.11 (0.43)	34.67 (7.26)	21.77 (1.27)	38.00 (8.99)
North China (Tianjin, TJ)	25.20 (1.26)	49.78 (7.04)	25.19 (1.91)	48.29 (2.65)
Northwest China (Lanzhou, LZ)	25.16 (1.45)	58.78 (9.12)	28.81 (4.50)	54.20 (12.15)
East China (Shanghai, SH)	26.25 (1.09)	60.30 (11.56)	28.94 (4.64)	66.37 (9.91)
Central China (Changsha, CS)	23.11 (2.09)	37.46 (6.50)	24.22 (3.53)	33.08 (6.61)
Southwest China (Yunnan, YN)	21.02 (1.20)	58.70 (5.27)	22.92 (13.81)	60.08 (5.64)
South China (Haikou, HK)	27.32 (0.72)	56.74 (4.49)	30.37 (0.88)	81.27 (6.06)

Mean (SD) values for indoor and outdoor temperature (°C) and relative humidity (%) are reported for seven study regions: Changchun (CC), Tianjin (TJ), Shanghai (SH), Changsha (CS), Lanzhou (LZ), Yunnan (YN), and Hong Kong (HK). Indoor measurements represent typical residential or survey environments, while outdoor values were obtained from local meteorological records during the study period.

Table S2. Percentage contribution of water from food and drinking fluids to total water intake across seven geographical regions in China.

Group	Region	Water from Food (%)	Drinking Fluids Water (%)	P-value
Overall	Southeast	43.7 (36.5, 51.4) ^{a}	56.3 (48.6, 63.5)	< 0.001
Overall	East	43.5 (37.6, 52.4) ^{a}	56.5 (47.6, 62.4)	< 0.001
Overall	Northeast	42.2 (37.4, 48.7) ^{a}	57.8 (51.3, 62.6)	< 0.001
Overall	North-central	41.7 (34.9, 49.7) ^{a}	58.3 (50.3, 65.1)	< 0.001
Overall	West	36.6 (30.5, 43.5) ^{b}	63.4 (56.5, 69.5)	< 0.001
Overall	Southwest	34.8 (23.7, 42.8) ^{b}	65.2 (57.2, 76.3)	< 0.001
Overall	South	34.4 (28.2, 40.0) ^{b}	65.6 (60.0, 71.8)	< 0.001
Male	Northeast	44.3 (38.5, 50.0) ^{a}	55.7 (50.0, 61.5)	< 0.001
Male	Southeast	43.7 (35.9, 52.8) ^{a}	56.3 (47.2, 64.1)	< 0.001
Male	East	40.6 (34.8, 51.1) ^{ab}	59.4 (48.9, 65.2)	< 0.001
Male	North-central	38.8 (32.8, 48.0) ^{ab}	61.2 (52.0, 67.2)	< 0.001
Male	South	35.9 (30.1, 41.7) ^{b}	64.1 (58.3, 69.9)	< 0.001
Male	Southwest	35.7 (24.6, 46.0) ^{b}	64.3 (54.0, 75.4)	< 0.001
Male	West	35.2 (28.9, 41.0) ^{b}	64.8 (59.0, 71.1)	< 0.001
Female	East	45.1 (39.3, 54.0) ^{b}	54.9 (46.0, 60.7)	< 0.001
Female	Southeast	43.7 (38.2, 50.4) ^{ab}	56.3 (49.6, 61.8)	< 0.001
Female	North-central	43.0 (38.1, 50.1) ^{ab}	57.0 (49.9, 61.9)	< 0.001
Female	Northeast	40.3 (36.2, 45.4) ^{ab}	59.7 (54.6, 63.8)	< 0.001
Female	West	37.9 (32.2, 45.3) ^{ad}	62.1 (54.7, 67.8)	< 0.001
Female	South	32.9 (26.5, 38.1) ^{c}	67.1 (61.9, 73.5)	< 0.001
Female	Southwest	32.7 (23.6, 40.2) ^{cd}	67.3 (59.8, 76.4)	< 0.001

Values are presented as median (25th, 75th percentiles). Comparisons were based on the percentage of water from food. The P-value indicates the overall significance among regions, as determined by the Kruskal–Wallis H test. Within each column and group (Overall, Male, or Female), values with different superscript letters (a–d) are significantly different ($P < 0.05$) according to Dunn's post-hoc test with Bonferroni correction. Regions sharing the same letter are not significantly different.

Table S3. Associations of demographic, psychosocial, lifestyle, and dietary factors with water from food (WFF) intake and its percentage contribution to total water intake among Chinese adults.

	**WFF (Male)**		**WFF(Female)**		**%WFF(Male)**		**%WFF(Female)**	
**Variables**	**β (95% CI)**	***p***	**β (95% CI)**	***p***	**β (95% CI)**	***p***	**β (95% CI)**	***p***
Age(years)	-1.742[-17.939, 14.454]	0.833	11.997[-2.241, 26.235]	0.098	-0.006[-0.013, 0.001]	0.08	-0.008[-0.014, -0.001]	0.028
Ethnicity (non-Han)	23.318[-38.13, 84.765]	0.456	-11.217[-65.137, 42.703]	0.683	0.012[-0.014, 0.039]	0.361	0.012[-0.013, 0.038]	0.341
Socioeconomical Tier (1.5)	43.628[-191.459, 278.714]	0.595	-33.658[-366.947, 299.631]	0.715	0.003[-0.248, 0.255]	0.963	-0.036[-0.219, 0.147]	0.495
Socioeconomical Tier (2)	-122.162[-383.069, 138.745]	0.223	-179.514[-545.987, 186.96]	0.172	-0.057[-0.334, 0.22]	0.485	-0.103[-0.303, 0.098]	0.159
PA Level (Moderate)	17.757[-54.876, 90.389]	0.631	-17.307[-69.949, 35.335]	0.519	0.009[-0.022, 0.04]	0.561	-0.005[-0.03, 0.02]	0.687
PA Level (High)	34.33[-33.616, 102.276]	0.321	5.425[-46.993, 57.844]	0.839	0.004[-0.025, 0.034]	0.777	-0.014[-0.039, 0.011]	0.264
SAS Score	1.427[-2.298, 5.151]	0.452	1.666[-2.429, 5.762]	0.424	0.002[0, 0.003]	0.038	0.001[-0.001, 0.003]	0.175
SDS Score	0.014[-3.568, 3.596]	0.994	-2.356[-6.184, 1.472]	0.227	-0.001[-0.002, 0.001]	0.378	-0.001[-0.003, 0.001]	0.343
PSQI Score	4.714[-4.207, 13.634]	0.298	-9.282[-17.208, -1.356]	0.022	0.001[-0.003, 0.005]	0.712	-0.002[-0.006, 0.002]	0.251
Daily Energy Intake(kcal/day)	0.230[0.17, 0.29]	<0.001	0.099[0.033, 0.165]	0.003	0.000[0.000, 0.000]	<0.001	0.000[0.000, 0.000]	0.497
Daily Salt Intake(g/day)	11.159[4.86, 17.458]	0.001	19.349[12.285, 26.412]	<0.001	0.000[-0.002, 0.003]	0.775	0.002[-0.001, 0.005]	0.235
High Fat Intake	-22.996[-91.078, 45.085]	0.507	44.755[-30.696, 120.206]	0.244	-0.027[-0.056, 0.003]	0.076	0[-0.036, 0.035]	0.985
High Protein Intake	-44.747[-106.205, 16.712]	0.152	54.506[1.965, 107.047]	0.042	-0.014[-0.041, 0.013]	0.317	-0.004[-0.029, 0.02]	0.722
High Carbohydrates Intake	346.953[255.599, 438.307]	<0.001	193.069[138.02, 248.118]	<0.001	0.146[0.107, 0.185]	<0.001	0.067[0.041, 0.093]	<0.001
Region Temperature (℃)	-4.273[-60.608, 52.062]	0.752	2.979[-60.537, 66.495]	0.854	-0.007[-0.056, 0.042]	0.573	0.007[-0.027, 0.041]	0.466
Region Temperature (%)	-1.69[-11.801, 8.421]	0.506	-3.503[-14.673, 7.667]	0.302	0[-0.008, 0.009]	0.846	-0.002[-0.008, 0.004]	0.224

β coefficients (95% confidence intervals) and *p*-values were estimated from multiple linear regression models stratified by gender, examining the associations of sociodemographic, psychological, and dietary factors with absolute water from food (WFF) intake and its percentage of total water intake (%WFF). Models were adjusted for age, ethnicity, socioeconomic tier, physical activity level, anxiety (SAS score), depression (SDS score), sleep quality (PSQI score), daily energy and salt intake, macronutrient composition, and regional temperature. Statistical significance was defined as $p < 0.05$.

Table S4. Associations of demographic, psychosocial, lifestyle, and dietary factors with water from food (WFF) intake and its percentage of total water intake (%WFF) stratified by age group among Chinese adults.

Region	Variables	WFF		%WFF	
		β (95% CI)	*p*	β (95% CI)	*p*
**18-19 years**	Gender (Female vs Male)	21.903[-26.154, 69.96]	0.371	0.006[-0.017, 0.028]	0.605
	Ethnicity (non-Han)	59.279[-1.284, 119.842]	0.055	0.027[-0.002, 0.055]	0.064
	Socioeconomical Tier (1.5)	-21.915[-687.093, 643.264]	0.913	-0.022[-0.205, 0.162]	0.731
	Socioeconomical Tier (2)	-247.71[-955.891, 460.471]	0.316	-0.06[-0.257, 0.137]	0.412
	PA Level (Moderate)	3.8[-57.943, 65.544]	0.904	0.009[-0.02, 0.038]	0.549
	PA Level (High)	5.167[-51.443, 61.777]	0.858	-0.001[-0.028, 0.026]	0.939
	SAS Score	3.266[-0.312, 6.844]	0.073	0.002[0, 0.004]	0.025
	SDS Score	-1.24[-4.697, 2.216]	0.481	0[-0.002, 0.001]	0.610
	PSQI Score	-6.332[-14.988, 2.323]	0.151	-0.004[-0.008, 0]	0.037
	Daily Energy Intake(kcal/day)	0.277[0.211, 0.343]	<0.0001	0[0, 0]	0.019
	Daily Salt Intake(g/day)	13.627[7.414, 19.839]	<0.0001	0[-0.003, 0.003]	0.944
	High Fat Intake	36.265[-36.641, 109.17]	0.329	-0.009[-0.043, 0.025]	0.610
	High Protein Intake	-93.369[-155.112, -31.626]	0.003	-0.034[-0.062, -0.005]	0.022
	High Carbohydrates Intake	187.576[116.729, 258.423]	<0.0001	0.101[0.068, 0.134]	<0.0001
	Region Temperature (℃)	-34.19[-163.986, 95.606]	0.387	0.006[-0.034, 0.045]	0.606
	Region Temperature (%)	3.408[-18.331, 25.147]	0.604	-0.003[-0.009, 0.004]	0.271
**20-21 years**	Gender (Female vs Male)	13.799[-37.429, 65.027]	0.597	0.024[0.001, 0.048]	0.042
	Ethnicity (non-Han)	-22.214[-84.44, 40.013]	0.483	-0.001[-0.03, 0.027]	0.918
	Socioeconomical Tier (1.5)	-41.088[-348.701, 266.525]	0.637	-0.026[-0.271, 0.219]	0.695
	Socioeconomical Tier (2)	-191.612[-519.321, 136.097]	0.134	-0.113[-0.379, 0.153]	0.214
	PA Level (Moderate)	23.789[-44.959, 92.537]	0.497	-0.003[-0.034, 0.029]	0.871

	PA Level (High)	45.778[-22.549, 114.105]	0.189	-0.001[-0.032, 0.031]	0.963
	SAS Score	-3.428[-8.147, 1.291]	0.154	-0.001[-0.003, 0.002]	0.650
	SDS Score	1.358[-2.947, 5.662]	0.536	0[-0.002, 0.001]	0.625
	PSQI Score	4.943[-4.443, 14.329]	0.301	0.003[-0.001, 0.007]	0.162
	Daily Energy Intake(kcal/day)	0.129[0.062, 0.197]	<0.0001	0[0, 0]	0.015
	Daily Salt Intake(g/day)	15.042[7.237, 22.846]	<0.0001	0.003[-0.001, 0.006]	0.118
	High Fat Intake	-55.623[-140.327, 29.082]	0.197	-0.038[-0.077, 0.001]	0.054
	High Protein Intake	71.58[11.392, 131.767]	0.020	-0.004[-0.032, 0.024]	0.795
	High Carbohydrates Intake	260.098[189.072, 331.123]	<0.0001	0.066[0.033, 0.098]	<0.0001
	Region Temperature (℃)	9.604[-53.813, 73.021]	0.542	-0.001[-0.047, 0.045]	0.944
	Region Temperature (%)	-2.49[-15.217, 10.237]	0.403	-0.001[-0.009, 0.008]	0.79
**22-25 years**	Gender (Female vs Male)	83.647[-9.776, 177.069]	0.079	0.041[0.003, 0.079]	0.036
	Ethnicity (non-Han)	23.318[-100.084, 146.721]	0.709	0.023[-0.027, 0.072]	0.365
	Socioeconomical Tier (1.5)	87.385[-108.668, 283.438]	0.379	0.007[-0.54, 0.555]	0.95
	Socioeconomical Tier (2)	18.467[-161.665, 198.599]	0.840	-0.031[-0.676, 0.615]	0.812
	PA Level (Moderate)	-83.667[-229.277, 61.944]	0.258	-0.025[-0.083, 0.033]	0.395
	PA Level (High)	-7.392[-153.127, 138.344]	0.920	-0.029[-0.088, 0.03]	0.335
	SAS Score	-4.09[-13.464, 5.284]	0.389	0.001[-0.003, 0.005]	0.518
	SDS Score	1.183[-8.387, 10.754]	0.807	0[-0.004, 0.004]	0.909
	PSQI Score	-3.396[-21.422, 14.631]	0.710	0[-0.008, 0.007]	0.933
	Daily Energy Intake(kcal/day)	0.152[0.024, 0.279]	0.020	0[0, 0]	0.203
	Daily Salt Intake(g/day)	12.201[-3.693, 28.095]	0.131	-0.002[-0.008, 0.005]	0.606
	High Fat Intake	29.006[-96.321, 154.333]	0.648	-0.019[-0.07, 0.032]	0.456
	High Protein Intake	78.103[-51.558, 207.763]	0.235	0.024[-0.029, 0.078]	0.365
	High Carbohydrates Intake	145.845[-13.918, 305.609]	0.073	0.028[-0.037, 0.093]	0.395
	Region Temperature (℃)	19.424[-21.237, 60.084]	0.346	0.003[-0.086, 0.093]	0.873

Region Temperature (%)	-6.993[-13.05, -0.936]	0.024	-0.002[-0.019, 0.015]	0.569

Values are β coefficients (95% CI) from multivariable linear regression models. WFF indicates absolute water intake from food (mL/day), and %WFF indicates the percentage of water from food relative to total water intake. Age groups were categorized as 18–19, 20–21, and 22–25 years. Variables included gender, ethnicity, socioeconomic tier, physical activity level, anxiety (SAS score), depression (SDS score), sleep quality (PSQI score), and daily intake of energy, salt, fat, protein, and carbohydrate. Statistically significant associations are defined as $p < 0.05$.
